# Estimating the monthly pan evaporation with limited climatic data in dryland based on the extended long short-term memory model enhanced with meta-heuristic algorithms

**DOI:** 10.1038/s41598-023-32838-4

**Published:** 2023-04-12

**Authors:** Tonglin Fu, Xinrong Li

**Affiliations:** 1 School of Mathematics and Statistics, LongDong University, Qingyang, 745000 China; 2 Shapotou Desert Research and Experiment Station, Northwest Institute of Eco-Environment and Resources, Chinese Academy of Sciences, Lanzhou, 730000 China

**Keywords:** Environmental sciences, Hydrology

## Abstract

Accurate estimation of evaporation is of great significance for understanding regional drought, and managing and applying limited water resources in dryland. However, the application of the traditional estimation approaches is limited due to the lack of required meteorological parameters or experimental conditions. In this study, a novel hybrid model was proposed to estimate the monthly pan Ep in dryland by integrating long short-term memory (LSTM) with grey wolf optimizer (GWO) algorithm and Kendall-τ correlation coefficient, where the GWO algorithm was employed to find the optimal hyper-parameters of LSTM, and Kendall-τ correlation coefficient was used to determine the input combination of meteorological variables. The model performance was compared to the performance of other methods based on the evaluation metrics, including root mean squared error (RMSE), the normalized mean squared error (NMSE), the mean absolute error (MAE), the mean absolute percentage error (MAPE), and Nash–Sutcliffe coefficient of efficiency (NSCE). The results indicated that the optimal input meteorological parameters of the hybrid Kendall-τ-GWO-LSTM models are the monthly average temperature, the minimum air temperature, the maximum air temperature, the minimum values of RMSE, NMSE, MAE, and MAPE are 38.28, 0.20, 26.62, and 19.96%, and the maximum NSCE is 0.89, suggesting that the hybrid Kendall-τ-GWO-LSTM exhibit better model performance than the other hybrid models. Thus, the hybrid Kendall-τ-GWO-LSTM model was highly recommended for estimating pan Ep with limited meteorological information in dryland. The present investigation provides a novel method to estimate the monthly pan Ep with limited meteorological variables in dryland by coupling a deep learning model with meta-heuristic algorithms and the data preprocessing techniques.

## Introduction

Evaporation (Ep) is a highly non-linear physical process, which is profoundly affected by meteorological parameters, including temperature, wind speed, precipitation, solar radiation, etc.^1,2^. As a main component of water balance, it plays an extremely important role in the global hydrological cycle^3–5^. Accurate estimation of evaporation by using is a significant issue in ecological management ^6–11^, especially in arid sand land, where the stability and sustainability of the artificially re-vegetated belts depend on the effective utilization of the limited available water resources^12,13^.

In general, the direct measurements method (e.g., Class A pan, Lysimeter group) is largely restricted due to the limitation of experimental conditions in dryland^14–16^, and the physically-based methods (e.g., Dalton model, FAO-56 Penman–Monteith method, etc.) have the drawbacks that the estimated results are very sensitive to the errors of parameters^17,18^, and the key meteorological factors(e.g., relative humidity, latent heat of evaporation, radiation) are sometimes difficult to be measured in the arid sand land^19,20^. Therefore, it is necessary to construct the data-driven models to estimate the Ep with less meteorological information.

Recently, various data-driven shallow machine learning (ML) models, e.g. artificial neural networks (ANN)^11,20^, radial basis function neural networks (RBFNN)^21^, multilayer artificial neural networks (MLNN)^2,22^, extreme learning machine (ELM)^2,15^, random forest (RF)^7^, support vector machine (SVM)^5,12,13,23^, etc., have been widely used to simulate Ep with incomplete meteorological variables. Those models have the excellent capability of simulating the non-linear relationships between the Ep and meteorological variables^24,25^. As the hyper-parameters of the ML models determine the estimated results and accuracy, meta-heuristic algorithms, including genetic algorithm (GA)^6,26,27^, particle swarm optimization algorithm (PSO)^1,28^, whale optimization algorithm(WOA)^2,12,29^, flower pollination algorithm (FPA)^2^, grey wolf optimizer algorithm (GWO) ^12,13^, etc., were employed to obtain the optimal hyper-parameters of ML models. In addition, the data preprocessing techniques, including Kendall-τ correlation coefficient^29,30^, and entropy weight^31^ were used to find the effective input combination of ML models. Literature review shows that shallow ML models hybridized with meta-heuristic algorithms and data preprocessing techniques, namely, hybrid model, have higher estimation accuracy than shallow ML models or physically-based methods^2,7,32^. Such models are recommended as the best choice for estimating Ep with limited meteorological information in different climate zones^8,12,13,33–35^.

Although shallow ML models hybridized appropriate meta-heuristic algorithms and data preprocessing techniques have proven potentially capable of estimating Ep in different regions^2,6,7,32–35^, the output of those hybrid models exists large error since the structure of shallow ML models cannot fully simulate the non-linear relationships between the meteorological parameters and Ep^11,13,19,36,37^. To improve the estimating accuracy, deep learning models (e.g. recurrent neural network (RNN)^36^, deep neural network (DNN)^37^, temporal convolution neural network(TCNN)^37^, long short-term memory (LSTM)^12,38^, etc.) were employed to estimate the Ep. Literature review shows that the deep learning models, especially LSTM, have better model performance than that of the other deep learning models and shallow ML models, and are demonstrated as an effective method for estimating Ep in different regions^12,36–38^. However, the setting of hyper-parameters of LSTM is subjective or depends on experience, which inevitably leads to a large estimating error. The hyper-parameters of LSTM, including the number of hidden layers (NHL), the number of hidden units (NHU), epochs (E), the mini-batch size (MBS), and learning rate (LR), directly determine the estimated results, whereas, few studies use meta-heuristic algorithms to optimize the hyper-parameters of LSTM for more precise estimation of Ep.

In this paper, two typical ML models, i.e. LSTM and SVM, were selected as main estimating modules, and two new meta-heuristic algorithms, including GWO and WOA, were employed to obtain the optimal hyper-parameters of ML models, and Kendallτ-correlation coefficient was employed to determine the input combinations of ML models. The proposed hybrid models, including Kendall-τ-GWO-SVM, Kendall-τ-WOA-SVM, Kendall-τ-GWO-LSTM, and Kendall-τ-WOA-LSTM, were employed to estimate the monthly pan Ep with limited meteorological information, and the superiority of the proposed models was tested by using the standard evaluation metrics. The aims of this study were (1) to provide a novel approach for monthly pan Ep estimation with limited meteorological variables; (2) to obtain more robust and precise estimating results by coupling LSTM with heuristic algorithms and data preprocessing technique; (3) to find the optimal and minimum meteorological parameters to be observed in the study area. Compared to previous studies^14–22,36–38^, the proposed models simultaneously account for data preprocessing and hyper-parameters optimization of deep learning models, and can be recommended as an effective method to estimate Ep with limited meteorological information in dryland.

## Materials and methods

### Case study

**Figure 1:**
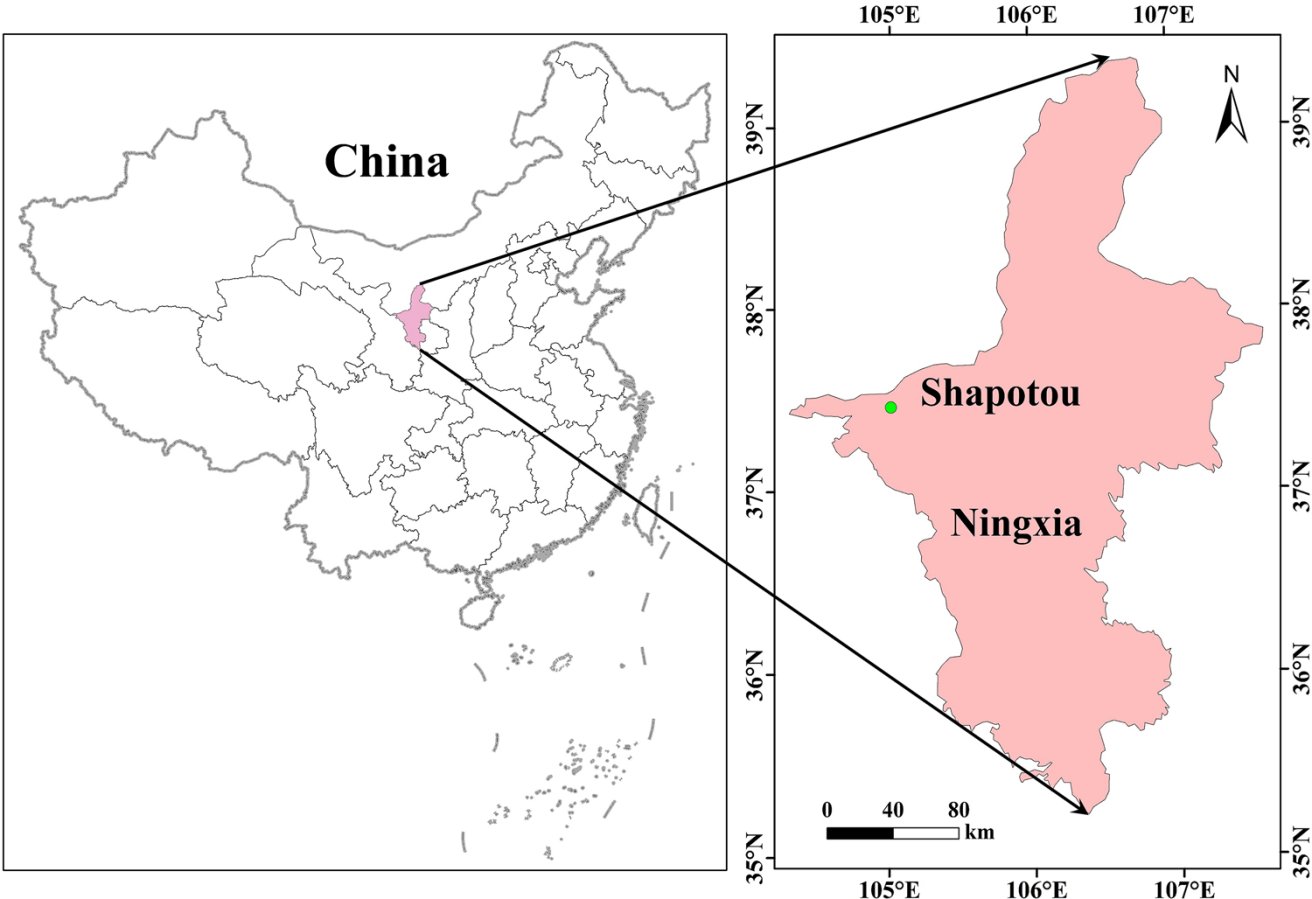
The location map of the study area (Using ArcGIS v. 10.8 software; Powered by ESRI “Environmental Systems Research Institute”, www.esri.com).

This study was conducted in the Shapotou (37°32′ N, 105°02′ E), Ningxia Hui Autonomous Region, China. Figure 1 shows the location map of the study area. This area is characterized by densely distributed trellis dunes, and it has the typical arid climate with scarce precipitation and huge evaporation, where the annual average precipitation is 180 mm and the annual average evaporation is 2520.4 mm^3^. To prevent the damage of sand erosion and promote regional ecological restoration, the artificial sand-binding vegetation belts were established in 1956a, and over subsequent years (1964a, 1981a and 1987a)^4,12,13^. It has been proved that revegetation is an effective approach for rehabilitation in arid sandy land^39^, ensuring the sustainability of artificial sand-binding vegetation under scarce precipitation and huge Ep is challenging for ecologists and land managers. Therefore, accurate estimation of Ep is of great theoretical and practical significance for understanding regional drought, managing and applying limited water resources, and determining the composition, structure, spatial distribution, and scale of artificial sand-binding vegetation.

### Data collection and analysis

The monthly meteorological variables needed to accomplish this study, including the monthly average temperature (T), the minimum air temperature (Tmin), the maximum air temperature (Tmax), the monthly precipitation (P), and the monthly average wind speed (WS), were compiled from the Shapotou Desert Research and Experiment Station from 1991 to 2018a. The data during 1991a–2010a was utilized as the training set, and the data during 2011a–2018a was used as the validation data set. Table 1 shows the minimum, maximum, mean, variance, skewness, and kurtosis of those measured meteorological parameters. As shown in Table 1, the average annual temperature in Shapotou during 1991–2018 was 10.8 ℃, with low-temperature and high-temperature extremes of − 26.2 ℃ and 40 ℃. The average monthly precipitation is 15.1 mm and the maximum precipitation is 117.3 mm. The average monthly Ep is 210 mm and the average monthly wind speed is 2.8 m/s. The probability distribution of all meteorological parameters is skewed.

**Table 1:** The statistical characteristics of the collected meteorological parameters.

Parameter	Data set	Min	Max	Mean	Variance	Skewness	Kurtosis
T (℃)	Training	9.8	26.7	10.69	113.25	0.19	1.36
	Testing	11.3	26.5	10.98	118.56	0.27	1.28
Tmin (℃)	Training	26.2	22.9	2.73	140.04	0.05	1.27
	Testing	23	15.4	2.11	132.00	0.09	1.31
Tmax (℃)	Training	5.5	39.9	25.10	96.33	0.43	1.14
	Testing	0	40	25.75	94.42	0.56	0.79
P (mm)	Training	0	99.2	14.67	397.47	2.00	3.92
	Testing	0	117.3	15.99	423.09	2.02	5.67
WS (m/s)	Training	1.2	4.4	2.93	0.43	0.14	0.38
	Testing	1.3	3.9	2.46	0.31	0.06	0.24

### Kendall-τ correlation coefficient

The Kendall-τ correlation coefficient is generally used to measure the correlation between two random variables without any assumption of population distribution. The definition of the Kendall-τ correlation coefficient is

1 $$\tau = \left( \begin{gathered} n \hfill \\ 2 \hfill \\ \end{gathered} \right)\sum\limits_{1 \le i < j \le n} {{\text{sgn}} ((a_{i} - a_{j} )(b_{i} - b_{j} ))}$$

with the sign function

2 $${\text{sgn}} (\alpha ) = \left\{ \begin{gathered} - 1,\alpha { < 0,} \hfill \\ 0,\alpha { = 0,} \hfill \\ 1,\alpha { > 0}{.} \hfill \\ \end{gathered} \right.$$

### Machine learning models

#### Long short-term memory (LSTM)

**Figure 2:**
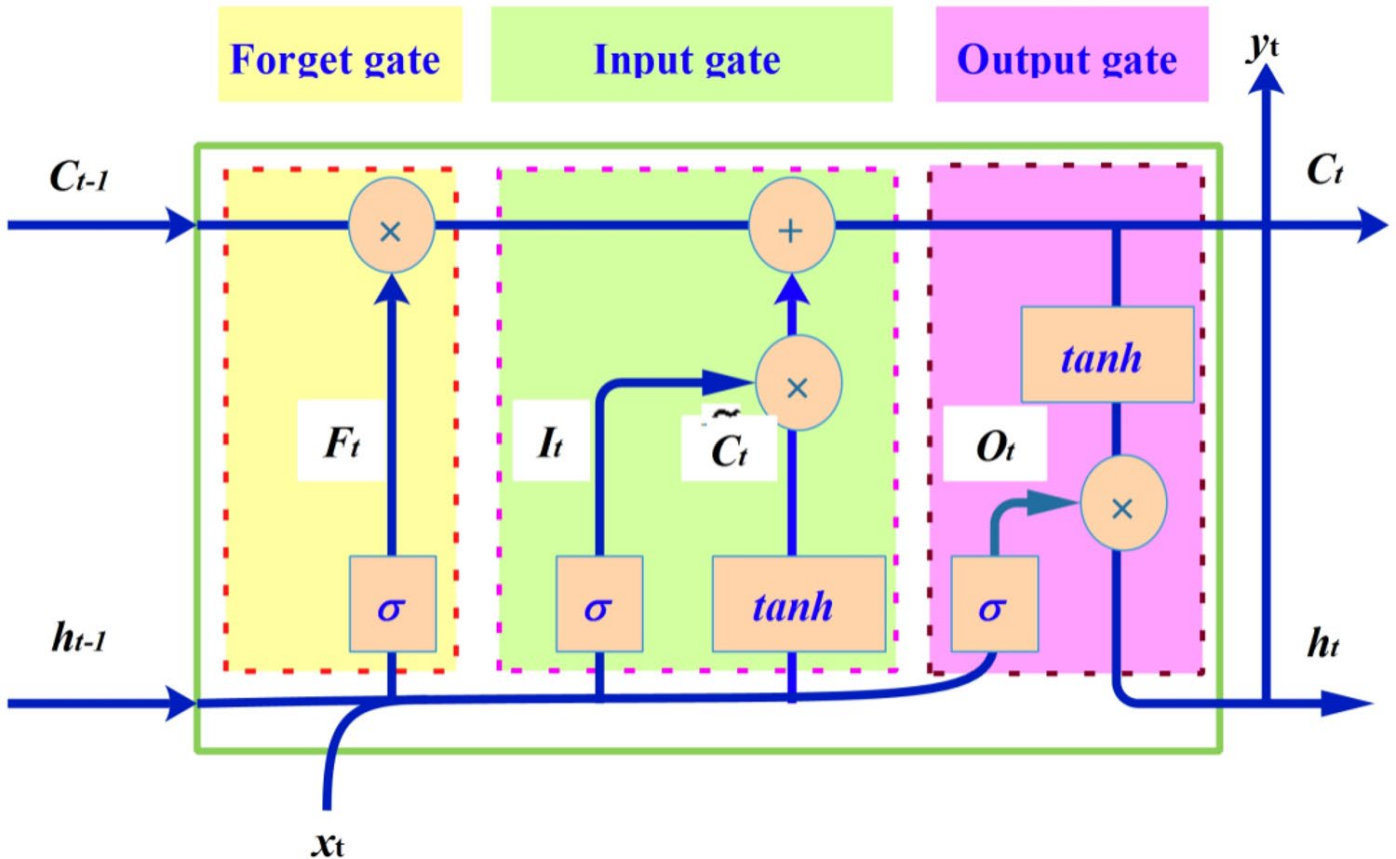
The internal structure of LSTM cell.

LSTM was designed to solve the gradient vanishing problem in RNN^40^. The significant difference between LSTM and RNN is that LSTM addresses the long-term dependency problems by adding repeating modules (cell) to store the information of the previous nodes^41^. Thus, LSTM was employed to estimate the evaporation in the study area. Figure 2 shows the internal structure of the LSTM cell, each memory cell consists forget gate $F_{t}$, input gate $I_{t}$, and output gate $O_{t}$, which are updated in the iterative process with

3 $$f_{t} = \sigma \left( {w_{hf} \cdot h_{t - 1 t} + w_{xf} \cdot x + b_{f} } \right)$$

4 $$i_{t} = \sigma \left( {w_{hi} \cdot h_{t - 1t} + w_{xi} \cdot x + b_{i} } \right)$$

5 $$\tilde{c}_{t} = \tanh \left( {w_{hc} \cdot h_{t - 1} + w_{xc} \cdot x_{t} + b_{c} } \right)$$

6 $$c_{t} = i_{t} * \tilde{c}_{t} + f_{t} * c_{t - 1}$$

7 $$o_{t} = \sigma \left( {w_{ho} \cdot h_{t - 1} + w_{xo} \cdot x_{t} + b_{o} } \right)$$

8 $$h_{t} = o_{t} * \tanh \left( {c_{t} } \right)$$

9 $$y_{t} = \sigma (w_{hy} \cdot h_{t} + b_{y} )$$

10 $$\sigma \left( x \right) = \left( {1 + e^{ - x} } \right)^{ - 1}$$

11 $$\tanh \left( x \right) = \frac{{e^{x} - e^{ - x} }}{{e^{x} + e^{ - x} }}$$

#### Support vector machine (SVM)

SVM is a typical shallow ML model that exhibited better model performance than other ML models to solve the nonlinear fitting problems by using kernel trick and Vapnik–Chervonenkis theory^23,42^. Thus, SVM was widely used to estimate Ep with limited meteorological variables in the field of hydrology^5,12,13,23,29^.

The regression coefficients are determined by solving the following problem

12 $$\begin{aligned} & \min obj\frac{1}{2}\left\| w \right\|^{2} + C\sum\limits_{i = 1}^{m} {(\xi_{i} } + \eta_{i} ) \\ & s.t.\left\{ \begin{gathered} \left\langle {w \cdot x_{i} } \right\rangle \alpha_{i} + b - y_{i} \le \varepsilon + \xi_{i} \hfill \\ y_{i} - \left\langle {w \cdot x_{i} } \right\rangle \alpha_{i} - b - \le \varepsilon + \eta_{i} \hfill \\ \xi_{i} ,\eta_{i} \ge 0,i = 1,2, \ldots n. \hfill \\ \end{gathered} \right. \\ \end{aligned}$$

The regression function $R(x)$ can be obtained by using Karush–Kuhn–Tucker’s method, which is

13 $$R(x,\alpha ,\alpha^{*} ) = \sum\limits_{i = 1}^{m} {\left( {\alpha_{i} { - }\alpha_{i}^{*} } \right)} k(x,x_{i} ) + b,$$

where $C > 0$ denote the penalty coefficient, $\xi_{i}$ and $\eta_{i}$ are the slack variable, $\alpha_{i}$ and $\alpha_{i}^{*}$ are Lagrange multiplications, respectively. The kernel function

14 $$k(x,x_{i} ) = \exp \left( { - G\parallel x - x_{i} \parallel } \right),$$

where $G = 0.5\sigma^{ - 2}$ denotes the radius of $k(x,x_{i} )$.

### Meta-heuristic algorithms

#### Grey wolf optimizer (GWO) algorithm

**Figure 3:**
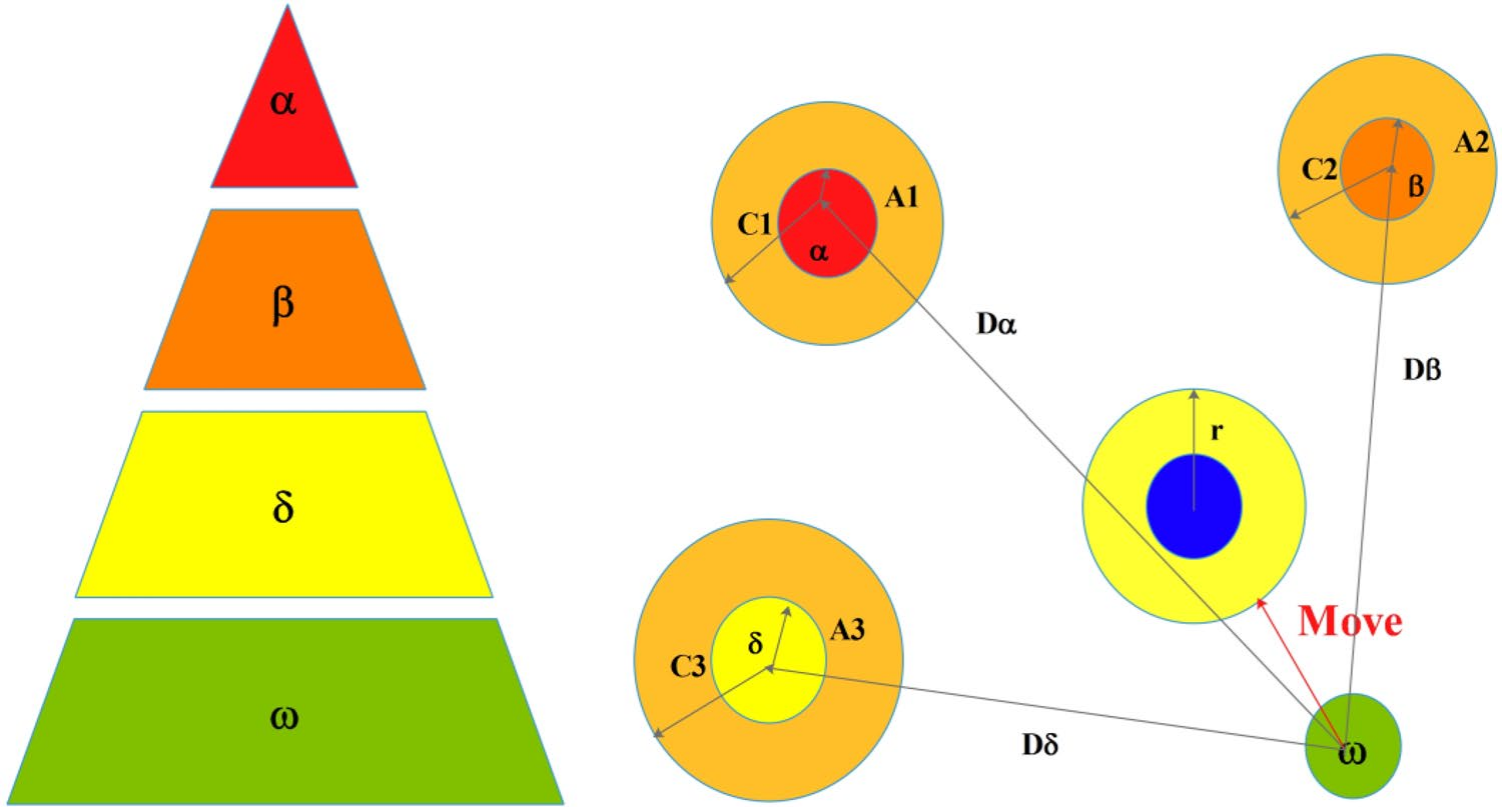
The population hierarchy of grey wolves and the position updating process of GWO.

GWO algorithm is a new meta-heuristic algorithm, the search process of GWO is inspired from the population hierarchy and predation behavior of the grey wolves^43^. Figure 3 shows the population hierarchy of grey wolves and the position updating process of GWO, where $\alpha ,\beta ,\delta$ and $\omega$ represents the grey wolves in the different hierarchical structures, and the dominance is decreased in sequence. In the simulation process, the distance and position vectors of different hierarchies are updated as

15 $$\overrightarrow {{{\mathbf{D}}_{\alpha } }} = \left| {\overrightarrow {{{\mathbf{C}}_{1} }} \overrightarrow {{{\mathbf{X}}_{\alpha }^{{^{p} }} (t)}} - \overrightarrow {{{\mathbf{X}}(t)}} } \right|,\overrightarrow {{{\mathbf{D}}_{\beta } }} = \left| {\overrightarrow {{{\mathbf{C}}_{2} }} \overrightarrow {{{\mathbf{X}}_{\beta }^{{^{p} }} (t)}} - \overrightarrow {{{\mathbf{X}}(t)}} } \right|,\overrightarrow {{{\mathbf{D}}_{\delta } }} = \left| {\overrightarrow {{{\mathbf{C}}_{3} }} \overrightarrow {{{\mathbf{X}}_{\delta }^{{^{p} }} (t)}} - \overrightarrow {{{\mathbf{X}}(t)}} } \right|,$$

16 $$\overrightarrow {{{\mathbf{X}}_{1} (t + 1)}} = \overrightarrow {{{\mathbf{X}}_{\alpha }^{p} (t)}} - \overrightarrow {{{\mathbf{A}}_{1} }} \overrightarrow {{{\mathbf{D}}_{\alpha } }} ,\overrightarrow {{{\mathbf{X}}_{2} (t + 1)}} = \overrightarrow {{{\mathbf{X}}_{\beta }^{p} (t)}} - \overrightarrow {{{\mathbf{A}}_{2} }} \overrightarrow {{{\mathbf{D}}_{\beta } }} ,\overrightarrow {{{\mathbf{X}}_{3} (t + 1)}} = \overrightarrow {{{\mathbf{X}}_{\delta }^{p} (t)}} - \overrightarrow {{{\mathbf{A}}_{3} }} \overrightarrow {{{\mathbf{D}}_{\delta } }} ,$$

17 $$\overrightarrow {{{\mathbf{X}}(t + 1)}} = \frac{{\left[ {\overrightarrow {{{\mathbf{X}}_{1} (t + 1)}} + \overrightarrow {{{\mathbf{X}}_{2} (t + 1)}} + \overrightarrow {{{\mathbf{X}}_{3} (t + 1)}} } \right]}}{3},$$

where the coefficient vectors $\overrightarrow {{\mathbf{A}}} = \overrightarrow {{{\varvec{\upalpha}}}} (2\overrightarrow {{{\mathbf{r}}_{1} }} - 1)$, and $\overrightarrow {{\mathbf{C}}} = 2\overrightarrow {{{\mathbf{r}}_{2} }}$, the random vectors $\overrightarrow {{{\mathbf{r}}_{1} }} ,\overrightarrow {{{\mathbf{r}}_{2} }} \in \left[ {0,1} \right]$, the attenuation factor $\overrightarrow {{{\varvec{\upalpha}}}}$ varies from 2 to 0. A more detailed description of GWO, we refer to Mirjalili et al.^43^ (Fig. 3).

**Figure 4:**
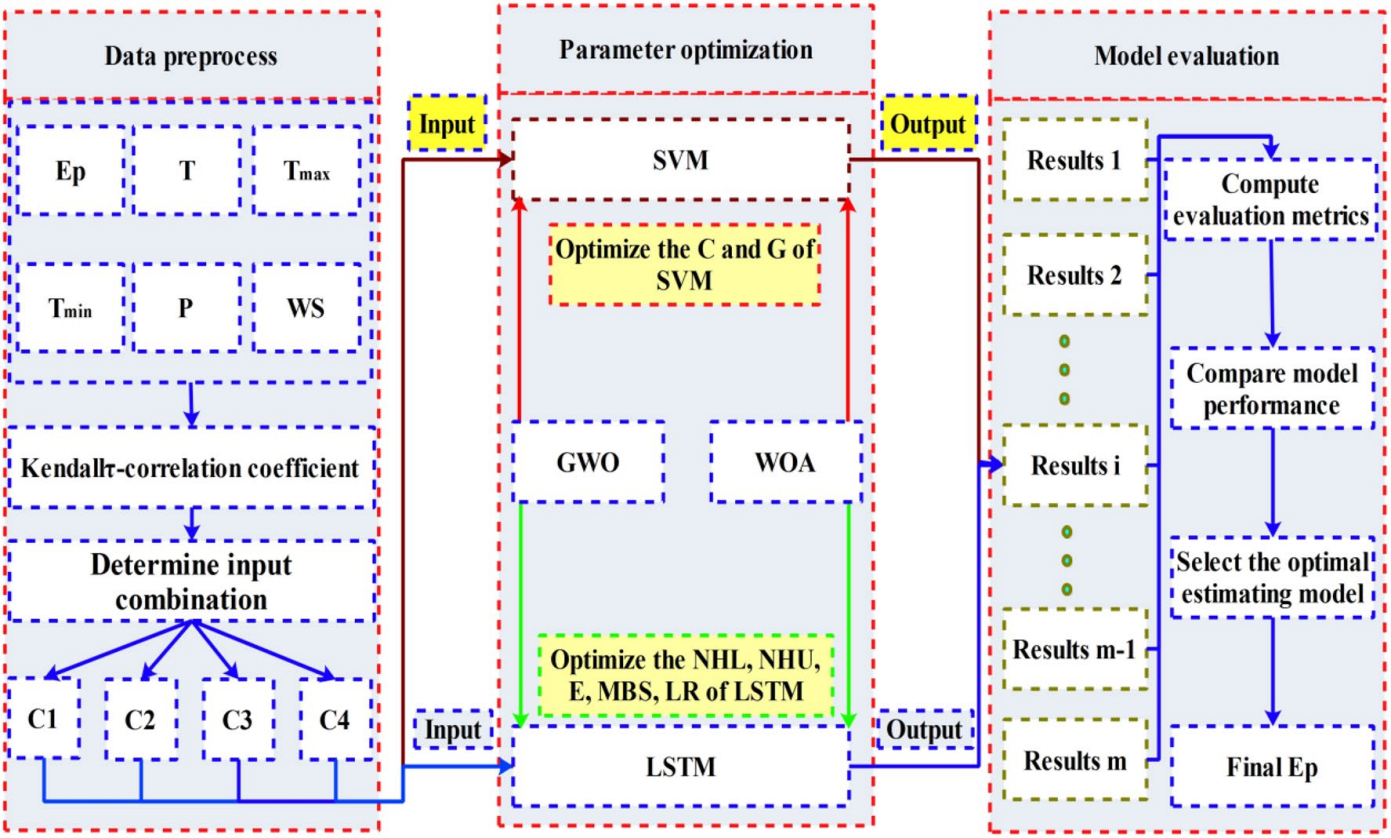
The flow chart of the estimating processes.

#### Whale optimization algorithm (WOA)

The WOA originated from the bubble-net feeding behavior of the humpback whale^44^. In the iterative process, the location vector of prey $\overrightarrow {{{\mathbf{X}}^{ * } (t)}}$ is regarded as the current best candidate solution, the humpback updates the positions vector **X**(*t*) along a spiral-shaped path, namely

18 $$\overrightarrow {{{\mathbf{X}}(t + 1)}} = \left\{ \begin{gathered} \overrightarrow {{{\mathbf{X}}^{ * } (t)}} - \overrightarrow {{\mathbf{A}}} \left| {C\overrightarrow {{{\mathbf{X}}^{ * } (t)}} - \overrightarrow {{{\mathbf{X}}(t)}} } \right|,\begin{array}{*{20}l} {} & {} & {} & {\begin{array}{*{20}c} {if} & {p < 0.5,} \\ \end{array} } \\ \end{array} \hfill \\ \left| {C\overrightarrow {{{\mathbf{X}}^{ * } (t)}} - \overrightarrow {{{\mathbf{X}}(t)}} } \right|e^{{bt}} \cos (2\pi l) + \overrightarrow {{{\mathbf{X}}^{ * } (t)}} ,\begin{array}{*{20}c} {if} & {p \ge 0.5,} \\ \end{array} \hfill \\ \end{gathered} \right.$$

the attenuation coefficient vectors

19 $$\overrightarrow {{\mathbf{A}}} = (2r - 1)\overrightarrow {{\mathbf{\alpha }}} ,$$

and

20 $$C = 2r ,$$

where b is a constant, *r* is a random variable in [0,1], the vector $\overrightarrow {{\mathbf{\alpha }}}$ decreases from 2 to 0, and *l* varies from 0 to 1, the random number *p* is used to judge whether the search process enters the bubble attack stage or performs the global search mechanism^44^.

### Hybrid models

In this study, the hybrid models, including Kendall-τ-GWO-SVM, Kendall-τ-WOA-SVM, Kendall-τ-GWO-LSTM, and Kendall-τ-WOA-LSTM, were proposed and employed to estimate the monthly pan Ep in the study area with incomplete meteorological information. It should be noted that Kendall-τ-WOA-LSTM denotes the LSTM coupled with the WOA algorithm and Kendall-τ correlation coefficient, the meaning of the Kendall-τ-GWO-SVM, Kendall-τ-WOA-SVM, Kendall-τ-GWO-LSTM models are similar to that of the Kendall-τ-WOA-LSTM. Figure 4 schematically illustrates the estimating processes in this study. As shown in Fig. 4, the estimating process includes three modules: the data pre-processing module, the parameters optimization module, and the model evaluation module, the main steps are as follows:

Step 1. The Kendall-τ correlation coefficient was employed to recognize the effective input variables of each ML model, and the training and testing data were normalized by using the min-max normalization method.

Step 2. SVM and LSTM were selected as the main estimating modular to achieve accurate estimate the evaporation in the study area.

Step 3. WOA and GWO were used to find the best penalty coefficient (C) and radius (G) of the SVM, and determine the optimal hyper-parameters of LSTM, including NHL, NHU, E, MBS, and LR, respectively.

Step 4. The root mean squared error (RMSE) was used to choose the best hybrid models with optimal hyper-parameters from 5 replications for each fixed meteorological parameter, and the optimal input meteorological parameters were determined according to the model performance.

Step 5. The estimated performance of the proposed models was compared by using the standard statistics metrics.

Step 6. The optimal estimating model was determined based on the evaluation results.

### Evaluation metrics

In this paper, the evaluation metrics, including RMSE^22^, the normalized mean squared error (NMSE)^12^, the mean absolute error (MAE)^9,13,22^, the mean absolute percentage error (MAPE)^14,22^, and Nash–Sutcliffe coefficient of efficiency (NSCE)^12,13^ were employed to assess the model performance. The definition of those evaluation indexes are as follows:

21 $$RMSE = \sqrt {\frac{{1}}{n}\sum\limits_{i = 1}^{n} {\left( {Ep_{i} - \widehat{Ep}_{i} } \right)^{2} } }$$

22 $$NMSE = \frac{{1}}{n}\sum\limits_{i = 1}^{n} {\left( {\frac{{Ep_{i} - \widehat{Ep}_{i} }}{{Ep_{i} }}} \right)^{2} }$$

23 $$MAE = \frac{{1}}{n}\sum\limits_{i = 1}^{n} {\left| {Ep_{i} - \widehat{Ep}_{i} } \right|}$$

24 $$MAPE = \frac{{1}}{n}\sum\limits_{i = 1}^{n} {\left| {\frac{{Ep_{i} - \widehat{Ep}_{i} }}{{Ep_{i} }}} \right|} \times 100\%$$

25 $$NSCE = 1 - \sum\limits_{i = 1}^{n} {\left( {Ep_{i} - \widehat{Ep}_{i} } \right)^{2} } /\sum\limits_{i = 1}^{n} {\left( {Ep_{i} - Ep} \right)^{2} }$$

where $Ep_{i}$ and $\widehat{Ep}_{i}$ denoted as the desired and actual outputs. It should be noted that RMSE, NMSE, MAE, and MAPE are generally employed to describe the error of the estimated results, those evaluation metrics approach 0 suggesting that the outputs of proposed models are close to the desired results. Thus, RMSE, NMSE, MAE, and MAPE are regarded as negative statistical metrics^12,13^^.^ NSCE can be employed to describe the model efficiency and measure the goodness of fit, NSCE close to 1 indicates the model has good fitness, thus, NSCE is regarded as positive evaluation metric^12,13^. The list of abbreviations used in this manuscript is shown in Table 2.

**Table 2:** List of abbreviations.

Abbreviation	Complete name	Abbreviation	Complete name
ANN	Artificial neural networks	NHL	Number of hidden layers
C	The penalty coefficient	NHU	Number of hidden units
DNN	Deep neural network	NSCE	Nash–Sutcliffe coefficient of efficiency
ELM	Extreme learning machine	NMSE	Normalized mean squared error
E	Epochs	NHL	Number of hidden layers
Ep	Evaporation	P	The monthly precipitation
FPA	Flower pollination algorithm	PSO	Particle swarm optimization algorithm
G	The radius of the kernel function	RBFNN	Radial basis function neural networks
GA	Genetic algorithm	RF	Random forest
GWO	Grey wolf optimizer	RMSE	Root mean squared error
LR	Learning rate	RNN	Recurrent neural network
LSTM	Long short-term memory	SVM	Support vector machine
MAE	Mean absolute error	T	The monthly average temperature
MAPE	Mean absolute percentage error	TCNN	Temporal convolution neural network
MBS	Mini-batch size	Tmin	The minimum air temperature
ML	Machine learning	Tmax	The maximum air temperature
MLNN	Multilayer artificial neural networks	WOA	Whale optimization algorithm

## Results

As mentioned above, the SVM and LSTM were regarded as the main modular to compute the monthly evaporation, respectively. To determine the input combination of ML models, the Kendall correlation coefficients between the meteorological variables, including T, Tmax, Tmin, P, WS, and Ep were calculated and shown in Table 3.

**Table 3:** The Kendall correlation coefficient between meteorological variables and Ep.

	T (°C)	Tmin (°C)	Tmax (°C)	P (mm)	WS (m/s)	Ep (mm)
T (°C)	1	0.840	0.851	0.526	0.270	0.731
Tmin (°C)	0.840	1	0.755	0.568	0.227	0.636
Tmax (°C)	0.851	0.755	1	0.503	0.289	0.725
P (mm)	0.526	0.568	0.503	1	0.175	0.386
WS (m/s)	0.270	0.227	0.289	0.175	1	0.418
Ep (mm)	0.731	0.636	0.725	0.386	0.418	1

Table 3 shows that T, Tmax, and Tmin have the highest correlation with evaporation, and WS and P have the next highest correlation, the Kendall correlation coefficients are 0.731, 0.725, 0.636, 0.418, and 0.386, respectively. With Kendall correlation coefficient greater than 0.5 as the threshold, T, Tmax, and Tmin were selected as the fixed input variables of all ML models, thus, the input meteorological variables combinations are C1 (T, Tmax, Tmin, P, WS), C2 (T, Tmax, Tmin, WS), C3 (T, Tmax, Tmin, P), and C4 (T, Tmax, Tmin). The input meteorological variables combinations, including C1, C2, C3, and C4, were input into the SVM and LSTM to estimate the monthly Ep, respectively. The input dimension of each ML model was the number of input variables.

GWO and WOA are new efficient meta-heuristic optimization techniques that inspired from the predation behavior of grey wolves and humpback whales^43,44^, respectively. At present, these two algorithms have been widely used to optimize the hyperparameters of shallow ML models, and show better ergodicity and global optimization capacity than other heuristic algorithms^12,13,29^. However, few studies using GWO or WOA to optimize deep learning models, especially finding the optimal hyperparameters of LSTM in the hydrological field. In this study, to overcome the defects of ML models sensitive to parameter selection, the heuristic algorithms (GWO and WOA) were employed to find the optimal hyper-parameters of SVM and LSTM, respectively. Table 4 shows the parameter setting of the proposed models.

**Table 4:** The parameters setting of the proposed models.

Model	Search agents	Maximum iterations	C	G	NHU	NHL	E	MBS	LR
Kendall-τ-WOA-SVM	5	100	[0.01, 1000]	[0.01, 1000]	_	_	_	_	_
Kendall-τ-GWO-SVM	5	100	[0.01, 1000]	[0.01, 1000]	_	_	_	_	_
Kendall-τ-GWO-LSTM	5	100	_	_	[1, 200]	[1, 200]	[1, 200]	[10, 100]	[0.001, 0.01]
Kendall-τ-WOA-LSTM	5	100	_	_	[1, 200]	[1, 200]	[1, 200]	[10, 100]	[0.001, 0.01]

As the randomness of some parameters in heuristic algorithms, the output of hybrid models was inconsistent. Thus, the relevant hyper-parameters and estimation accuracy of each hybrid model were recorded from five replications. Tables 5, 6, 7, 8 show the optimal parameters of each proposed models obtained by the heuristic algorithm in the training stage, and the evaluation indexes are also listed (The estimating results of the proposed models with different input with different input combinations and optimal hyper-parameters are shown in Supplementary File). It should be noted that the optimal hyper-parameters and evaluation metrics of those hybrid models with different input combinations are marked in bold. E.g., Table 5 shows that the optimal hyper-parameters of the hybrid Kendall-τ-GWO-SVM model in the training stage with different combinations are: C1 (C = 214.76, G = 0.001), C2 (C = 700.49, G = 0.014), C3 (C = 339.44, G = 0.013), and C4 (C = 434.08, G = 0.063), the minimum MAPE with the input combinations C1, C2, C3, and C4 in the testing stage are 30.71%, 30.34%, 26.97%, 32.32%, and the maximum NSCE are 0.74, 0.72, 0.77, 0.76, the results of other evaluation metrics are omitted. Table 7 shows that the optimal hyper-parameters of the hybrid Kendall-τ-GWO-LSTM model with input combinations C1-C4 in the training stage are: C1 (NHL = 6, NHU = 15, E = 96, MBS = 24, LR = 0.003), C2 (NHL = 10, NHU = 51, E = 39, MBS = 44, LR = 0.008), C3(NHL = 52, NHU = 89, E = 68, MBS = 16, LR = 0.007) and C4(NHL = 47, NHU = 93, E = 57, MBS = 20, LR = 0.005), the minimum MAPE with the input combinations C1, C2, C3 and C4 in testing stage are 26.17%, 27.97%, 23.03%, 19.96%, and the maximum NSCE are 0.81, 0.80, 0.86, 0.89, respectively. The meanings of the results in Tables 6 and 8 are similar to that of Tables 5 and 7.

**Table 5:** The optimal hyper-parameters and model performance of Kendall-τ-GWO-SVM.

Input variables	Kendall-τ-GWO-SVM				Training					Testing		
	C	G	RMSE	NMSE	MAE	MAPE	NSCE	RMSE	NMSE	MAE	MAPE	NSCE
T, Tmax, Tmin, P, WS	139.95	0.001	30.65	0.05	26.23	16.33%	0.94	55.25	0.17	43.50	30.73%	0.7348
	108.19	0.002	30.59	0.05	26.15	16.15%	0.94	55.38	0.17	43.63	30.76%	0.7335
	25.07	0.002	31.07	0.05	24.43	13.35%	0.94	55.68	0.19	45.70	33.90%	0.7306
	521.89	0.001	30.98	0.05	26.77	**17.93%**	0.94	55.39	0.17	43.27	30.76%	0.7334
	**214.76**	**0.001**	**30.72**	**0.05**	**26.34**	16.81%	**0.94**	**55.18**	**0.17**	**43.35**	**30.71%**	**0.7355**
T, Tmax, Tmin, WS	820.38	0.117	30.56	0.09	26.72	14.96%	0.94	56.61	0.24	48.58	37.96%	0.7215
	604.49	0.121	30.60	0.09	26.78	**15.01%**	0.94	56.59	0.24	48.63	38.11%	0.7217
	**700.49**	**0.014**	**29.51**	**0.05**	**25.13**	15.58%	**0.94**	**56.59**	**0.15**	**44.41**	**30.34%**	**0.7217**
	13.70	0.121	30.60	0.09	26.78	15.01%	0.94	56.59	0.24	48.63	38.11%	0.7217
	159.10	0.121	30.60	0.09	26.78	15.01%	0.94	56.59	0.24	48.63	38.11%	0.7217
T, Tmax, Tmin, P	238.45	0.013	30.25	0.05	25.70	**16.20%**	0.94	51.85	0.14	39.79	26.98%	0.7664
	893.50	0.014	30.14	0.05	25.56	15.99%	0.94	51.84	0.14	39.84	27.11%	0.7665
	**339.44**	**0.013**	**30.27**	**0.05**	**25.72**	16.24%	**0.94**	**51.86**	**0.14**	**39.79**	**26.97%**	**0.7665**
	162.01	0.014	30.16	0.05	25.59	**16.07%**	0.94	51.86	0.14	39.82	27.06%	0.7663
	420.53	0.014	30.15	0.05	25.58	16.06%	0.94	51.86	0.14	39.82	27.05%	0.7663
T, Tmax, Tmin	95.90	0.066	29.96	0.06	25.91	14.19%	0.94	52.91	0.19	43.68	32.80%	0.7568
	**434.08**	**0.063**	**29.88**	**0.06**	**25.81**	14.19%	**0.94**	**52.90**	**0.18**	**43.52**	**32.32%**	**0.7569**
	95.98	0.065	29.94	0.06	25.88	**14.19%**	0.94	52.90	0.19	43.65	32.70%	0.7568
	234.43	0.063	29.89	0.06	25.83	14.19%	0.94	52.90	0.18	43.55	32.40%	0.7568
	363.43	0.064	29.93	0.06	25.86	14.20%	0.94	52.91	0.18	43.61	32.56%	0.7568

The optimal hyper-parameters and evaluation metrics of the hybrid Kendall-τ-GWO-SVM model are marked in bold.

**Table 6:** The optimal hyper-parameters and model performance of Kendall-τ-WOA-SVM.

Input variables	Kendall-τ-WOA-SVM		Training					Testing				
	C	G	RMSE	NMSE	MAE	MAPE	NSCE	RMSE	NMSE	MAE	MAPE	NSCE
T, Tmax, Tmin, P, WS	**98.94**	**0.002**	**30.56**	**0.05**	**26.13**	**16.12%**	**0.94**	**55.42**	**0.17**	**43.64**	**30.72%**	**0.73**
	23.46	0.002	31.16	0.05	24.41	13.27%	0.94	55.59	0.20	45.69	34.02%	0.73
	86.60	0.002	30.49	0.05	26.04	16.02%	0.94	55.51	0.17	43.74	30.72%	0.73
	24.79	0.002	31.16	0.05	24.42	13.28%	0.94	55.56	0.20	45.66	33.97%	0.73
	109.48	0.002	30.61	0.05	26.30	16.74%	0.94	55.84	0.17	43.80	30.81%	0.73
T, Tmax, Tmin, WS	59.41	0.121	30.60	0.09	26.78	15.01%	26.78	56.59	0.24	48.63	38.11%	0.72
	155.14	0.121	30.60	0.09	26.78	**15.01%**	26.78	56.59	0.24	48.63	38.11%	0.72
	603.94	0.116	30.51	0.09	26.67	14.93%	26.67	56.61	0.24	48.54	37.86%	0.72
	1.33	0.145	31.02	0.09	27.23	15.34%	27.23	56.78	0.26	49.23	39.27%	0.72
	**2.50**	**0.006**	**33.58**	**0.05**	**25.86**	13.50%	**25.86**	**55.03**	**0.20**	**46.11**	**34.47%**	**0.74**
T, Tmax, Tmin, P	7.65	0.013	31.30	0.06	25.35	14.06%	0.94	51.49	0.17	42.09	31.46%	0.77
	217.58	0.015	30.11	0.05	25.53	15.92%	0.94	51.87	0.14	39.88	27.20%	0.77
	**145.35**	**0.013**	**30.27**	**0.05**	**25.72**	**16.24%**	**0.94**	**51.86**	**0.14**	**39.79**	**26.97%**	**0.77**
	176.67	0.015	30.06	0.05	25.46	15.87%	0.94	51.87	0.14	39.87	27.20%	0.77
	696.63	0.018	29.91	0.05	25.34	15.60%	0.94	51.87	0.15	40.08	27.65%	0.77
T, Tmax, Tmin	3.97	0.136	30.33	0.08	26.44	14.65%	0.94	53.05	0.24	44.98	36.61%	0.76
	35.08	0.062	29.88	0.06	25.80	14.19%	0.94	52.91	0.18	43.50	32.2z3%	0.76
	**65.19**	**0.002**	**32.18**	**0.05**	**25.73**	14.18%	**0.93**	**51.28**	**0.13**	**41.54**	**28.38%**	**0.76**
	160.04	0.062	29.89	0.06	25.82	**14.19%**	0.94	52.91	0.18	43.53	32.31%	0.76
	191.94	0.062	29.89	0.06	25.82	14.19%	0.94	52.91	0.18	43.55	32.37%	0.76

The optimal hyper-parameters and evaluation metrics of the hybrid Kendall-τ-WOA-SVM model are marked in bold.

**Table 7:** The optimal hyper-parameters and model performance of Kendall-τ-GWO-LSTM.

Input variables	Kendall-τ-GWO-LSTM					Training					Testing				
	NHU	NHL	E	MBS	LR	RMSE	NMSE	MAE	MAPE	NSCE	RMSE	NMSE	MAE	MAPE	NSCE
T, Tmax, Tmin, P, WS	**6**	**15**	**96**	**24**	**0.003**	**28.77**	**0.03**	**22.17**	**12.42%**	**0.95**	**46.72**	**0.39**	**32.97**	**26.17%**	**0.81**
	58	42	51	21	0.002	31.67	0.03	24.27	14.55%	0.94	46.25	0.40	34.11	27.00%	0.81
	37	31	48	39	0.002	33.87	0.03	26.19	16.20%	0.93	47.83	0.43	36.91	30.20%	0.80
	87	57	48	33	0.007	27.85	0.02	20.94	11.12%	0.95	48.79	0.30	37.20	27.35%	0.79
	52	32	37	16	0.007	34.23	0.03	26.10	14.58%	0.93	50.02	0.41	37.41	28.62%	0.78
T, Tmax, Tmin, WS	24	29	60	21	0.002	30.13	0.03	22.66	11.99%	0.94	48.99	0.40	37.15	28.94%	0.79
	27	36	32	49	0.003	32.57	0.04	24.64	13.06%	0.93	50.70	0.46	37.64	29.55%	0.78
	**10**	**51**	**39**	**44**	**0.008**	**39.23**	**0.04**	**30.09**	19.20%	**0.90**	**48.28**	**0.50**	**33.49**	**27.97%**	**0.80**
	21	35	50	28	0.006	31.74	0.03	23.98	13.44%	0.94	51.90	0.45	37.53	29.76%	0.77
	38	59	31	42	0.007	31.20	0.03	23.74	**13.39%**	0.94	50.50	0.42	39.08	31.49%	0.78
T, Tmax, Tmin, P	23	58	57	49	0.005	34.46	0.03	26.92	16.40%	0.92	41.63	0.31	32.34	25.32%	0.85
	50	22	79	52	0.007	29.23	0.03	21.83	11.35%	0.95	40.51	0.29	30.05	24.73%	0.86
	**52**	**89**	**68**	**16**	**0.007**	**32.73**	**0.03**	**25.67**	**15.62%**	**0.93**	**40.42**	**0.21**	**31.12**	**23.03%**	**0.86**
	26	39	81	37	0.004	32.09	0.03	24.77	14.71%	0.93	41.46	0.31	31.12	24.48%	0.85
	82	42	25	45	0.008	42.49	0.05	33.51	25.46%	0.88	43.62	0.27	33.81	25.36%	0.83
T, Tmax, Tmin	68	96	64	21	0.009	27.27	0.03	19.80	11.06%	0.95	35.60	0.19	26.81	21.69%	0.87
	38	55	38	46	0.008	34.65	0.03	26.90	15.99%	0.92	37.47	0.26	27.81	21.48%	0.88
	62	64	67	47	0.007	30.45	0.03	22.56	**11.88%**	0.94	38.99	0.21	30.39	23.66%	0.87
	**47**	**93**	**57**	**20**	**0.005**	**37.34**	**0.03**	**28.16**	15.25%	**0.91**	**38.28**	**0.20**	**26.62**	**19.96%**	**0.89**
	83	69	67	56	0.006	29.68	0.03	22.14	12.13%	0.94	38.45	0.28	28.29	22.77%	0.87

The optimal hyper-parameters and evaluation metrics of the hybrid Kendall-τ-GWO-LSTM model are marked in bold.

**Table 8:** The optimal hyper-parameters and model performance of Kendall-τ-WOA-LSTM.

Input variables	Kendall-τ-WOA-LSTM					Training					Testing				
	NHU	NHL	E	MBS	LR	RMSE	NMSE	MAE	MAPE	NSCE	RMSE	NMSE	MAE	MAPE	NSCE
T, Tmax, Tmin, P, WS	42	21	73	26	0.003	27.85	0.02	20.93	12.23%	0.95	50.57	0.38	38.62	30.29%	0.78
	44	98	42	38	0.007	31.41	0.03	24.44	17.80%	0.94	53.42	0.35	39.55	29.37%	0.75
	**96**	**59**	**11**	**34**	**0.009**	**57.95**	**0.06**	**42.98**	**22.16%**	**0.79**	**45.52**	**0.45**	**31.69**	**28.28%**	**0.82**
	26	12	57	23	0.003	28.98	0.03	21.64	12.34%	0.95	52.93	0.42	38.48	28.44%	0.76
	54	93	39	21	0.009	28.41	0.03	21.69	11.72%	0.95	51.92	0.27	39.85	29.64%	0.77
T, Tmax, Tmin, WS	48	56	50	39	0.004	26.58	0.02	20.31	11.50%	0.95	62.76	0.45	48.16	35.30%	0.66
	70	89	24	33	0.006	34.02	0.05	25.49	13.52%	0.93	54.72	0.45	41.26	31.11%	0.74
	93	91	14	42	0.008	34.67	0.05	27.08	14.55%	0.92	54.93	0.45	43.83	33.91%	0.74
	**78**	**38**	**51**	**32**	**0.004**	**26.89**	**0.02**	**20.53**	**11.93%**	**0.95**	**54.33**	**0.47**	**39.50**	**30.87%**	**0.74**
	68	85	26	23	0.002	35.01	0.07	27.36	14.87%	0.92	53.91	0.50	42.18	33.67%	0.75
T, Tmax, Tmin, P	60	57	65	31	0.003	31.73	0.03	24.23	15.17%	0.94	42.88	0.33	32.68	24.84%	0.84
	37	71	71	50	0.005	29.13	0.03	21.94	12.08%	0.95	42.59	0.30	31.74	24.44%	0.84
	**57**	**25**	**74**	**28**	**0.008**	**29.31**	**0.03**	**22.45**	**12.63%**	**0.95**	**41.02**	**0.26**	**30.45**	**22.87%**	**0.85**
	31	60	53	38	0.007	32.54	0.03	24.87	13.07%	0.93	43.48	0.25	34.10	25.93%	0.84
	46	96	92	46	0.007	23.22	0.02	18.12	11.34%	0.97	44.77	0.21	35.31	25.18%	0.83
T, Tmax, Tmin	28	74	58	32	0.008	35.18	0.04	25.83	13.23%	0.92	50.20	0.27	37.67	25.86%	0.78
	81	92	76	23	0.006	25.90	0.03	19.33	11.46%	0.96	38.04	0.22	27.68	20.99%	0.87
	99	83	99	36	0.005	20.32	0.02	15.69	9.10%	0.97	43.39	0.19	34.03	25.07%	0.84
	**63**	**76**	**46**	**29**	**0.005**	**34.53**	**0.03**	**26.53**	**15.52%**	**0.92**	**37.69**	**0.24**	**28.11**	**21.30%**	**0.88**
	93	59	74	19	0.008	27.87	0.03	20.59	12.41%	0.95	42.99	0.28	32.50	24.35%	0.84

The optimal hyper-parameters and evaluation metrics of the hybrid Kendall-τ-WOA-LSTM model are marked in bold.

**Figure 5:**
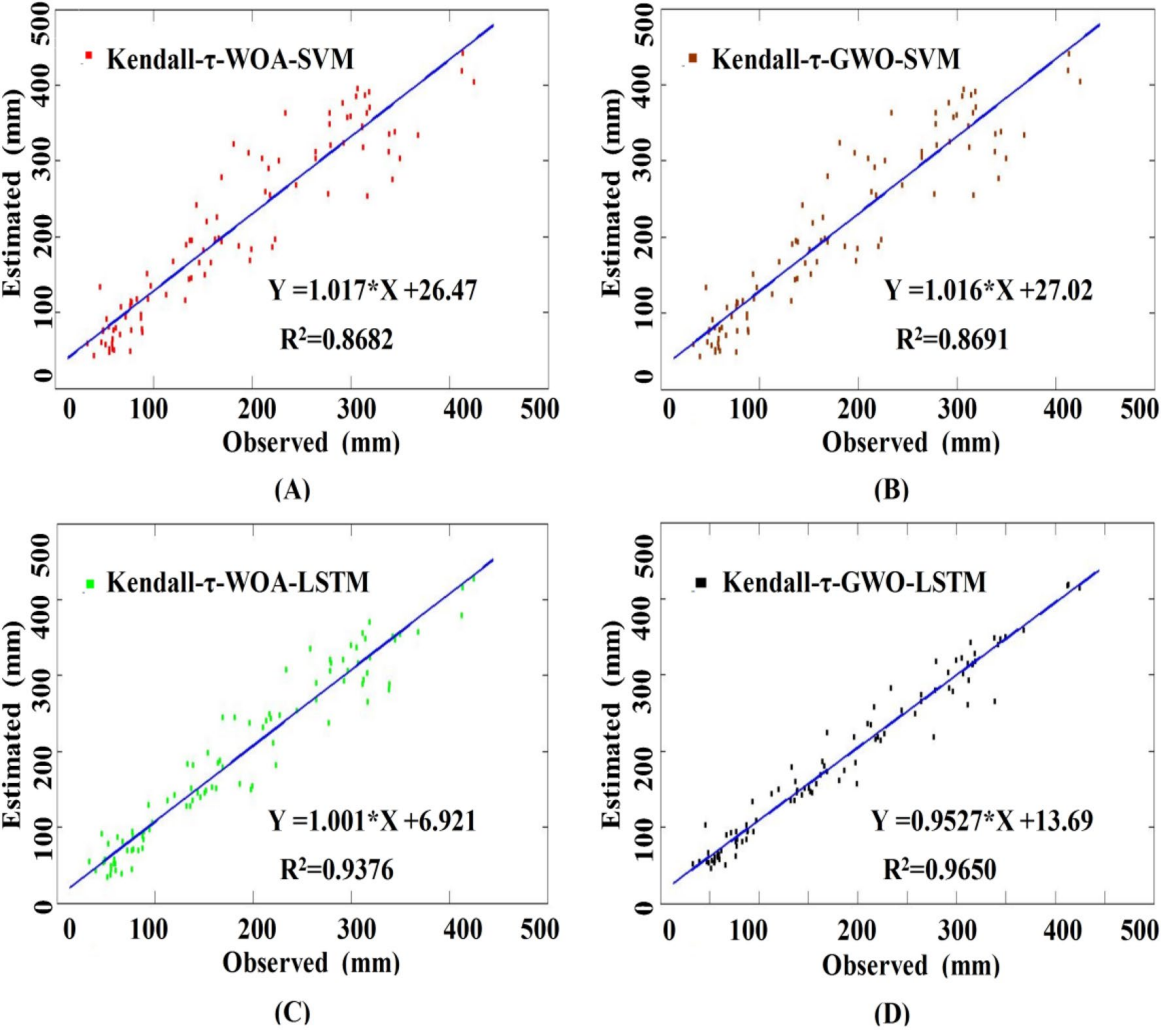
The scatter plots of the observed and estimated results of the proposed models. The blue line inside each panel denotes the fitted line between the observed and estimated results with the coefficient of determination (R^2^). (**A**) The results of the hybrid Kendall-τ-WOA-SVM model with C = 339.44 and G = 0.013. (**B**) The results of the hybrid Kendall-τ-GWO-SVM model with C = 145.35 and G = 0.013. (**C**) The results of the hybrid Kendall-τ-WOA-LSTM model with NHL = 63, NHU = 76, E = 46, MBS = 29, and LR = 0.005. (**D**) The results of the hybrid Kendall-τ-GWO-LSTM model with NHL = 47, NHU = 93, E = 57, MBS = 20, and LR = 0.005.

**Figure 6:**
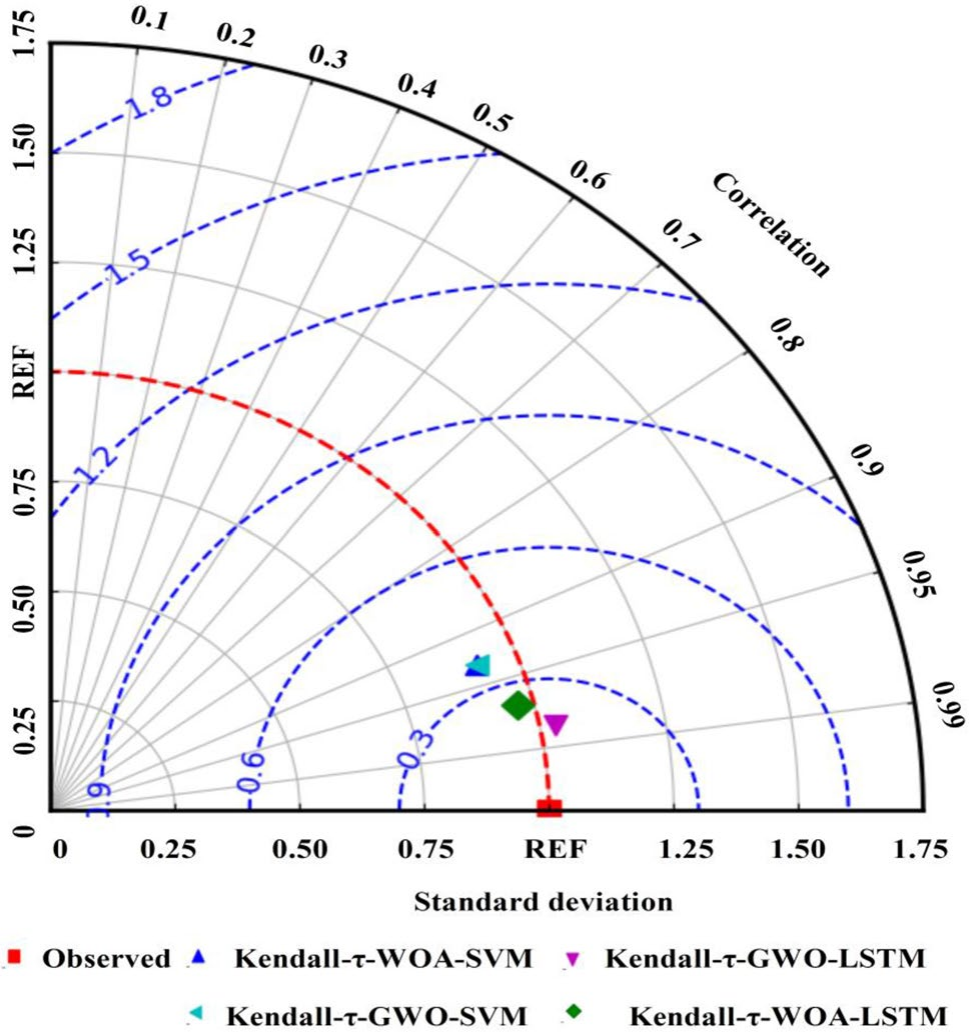
Taylor diagrams of the hybrid Kendall-τ-WOA-SVM, Kendall-τ-GWO-SVM, and Ksendall-τ-WOA-LSTM models with optimal hyper-parameters and input combinations.

The scatter plots of the desired and actual outputs of each model with optimal hyper-parameters and input combinations are shown in Fig. 5. As shown in Fig. 5, the hybrid Kendall-τ-GWO-SVM, Kendall-τ-WOA-SVM, Kendall-τ-GWO-LSTM, and Kendall-τ-WOA-LSTM models can be used to compute the monthly Ep and achieve high computing accuracy with the limited meteorological information, the coefficients of the regression lines are all greater than 1 except for that of the hybrid Kendall-τ-GWO-LSTM model, suggesting that the hybrid Kendall-τ-WOA-SVM, Kendall-τ-GWO-SVM, and Kendall-τ-WOA-LSTM models overestimated the monthly Ep, and the hybrid Kendall-τ-GWO-LSTM model underestimated the monthly Ep to a certain extent. To further compare the model performance of the hybrid Kendall-τ-WOA-SVM, Kendall-τ-GWO-SVM, Kendall-τ-WOA-LSTM, and Kendall-τ-GWO-LSTM models, the Taylor diagram is illustrated in Fig. 6. Taylor diagram shows the standard deviation, RMSE,and Pearson correlation coefficient on a two-dimensional chart, which provides an intuitive way to compare the model performance and reflects the simulation capability of the proposed models^10,11,18,35^. On the whole, Fig. 6 shows that the hybrid Kendall-τ-GWO-SVM model has higher Pearson correlation coefficient and lesser standard deviation and RMSE than that of the hybrid Kendall-τ-WOA-SVM, Kendall-τ-GWO-SVM, and Kendall-τ-WOA-LSTM models, indicating that the hybrid Kendall-τ-GWO-LSTM has superior performance than that of the other hybrid models.

## Discussion

The accuracies of the proposed models are determined by the different input combinations of meteorological variables, finding the optimal input combination of ML models can effectively improve the estimating accuracy. As shown in Tables 5, 6, 7, 8, the computing accuracies present different trends with different input meteorological variables. Taking the hybrid Kendall-τ-GWO-SVM model as an example, when the input meteorological variables are T, Tmax, Tmin, P, WS, the ranges of MAE, MAPE, RMSE, NMSE, and NSCE are [43.27, 45.70], [30.71%, 33.90%], [55.18, 55.68], [0.17, 0.19], and [0.73,0.74], respectively; When the input meteorological variables are T, Tmax, Tmin, and P, the ranges of MAE, MAPE, RMSE, and NMSE are [39.79, 39.84], [26.97%, 27.11%], [51.84, 51.86], [0.14, 0.14], and the maximum NSCE is 0.77, respectively. Thus, the computing accuracies of Kendall-τ-GWO-SVM were significantly improved when the input meteorological variables were optimized.

The statistical metrics in Tables 5, 6, 7, 8 show that RMSE, NMSE, MAE, and MAPE are not necessarily consistent with each other, which will lead to confusion if a different evaluation index is selected as a main benchmark to evaluate the model performance or find the optimal parameters of proposed models. Since MAPE and NSCE are two dimensionless quantities, the results of these two metrics are relatively more stable than the other evaluation indexes^12,13^. Thus, MAPE and NSCE were employed to determine the optimal input combination in this study (The discussion of other evaluation metrics is similar). As shown in Tables 5 and 6, the optimal and minimum input meteorological parameters of the hybrid Kendall-τ-GWO-SVM and Kendall-τ-WOA-SVM models are T, Tmax, Tmin, and P, the minimum MAPE is 26.97%, and the maximum NSCE is 0.77 from five replications. Tables 7 and 8 show that the optimal input meteorological parameters of the hybrid Kendall-τ-GWO-LSTM and Kendall-τ-WOA-LSTM models are T, Tmax, and Tmin, the minimum MAPE and the maximum NSCE of the hybrid Kendall-τ-GWO-LSTM model are 19.96% and 0.89; As for the hybrid Kendall-τ-WOA-LSTM model, the minimum MAPE and the maximum NSCE are 21.30% and 0.88. On the whole, the hybrid Kendall-τ-GWO-LSTM and Kendall-τ-WOA-LSTM models have outperformed the hybrid Kendall-τ-GWO-SVM and Kendall-τ-WOA-SVM models, and need fewer meteorological parameters to be observed.

To test whether there is a significant difference in the estimation accuracy of the proposed models under the same input combination, Kruskal–Wallis (K–W) test was performed on MAE, MAPE, NMSE, RMSE, and NSCE in the validation stage. K–W test is a non-parametric test method that does not need to assume that the variables to be tested obey normal distribution^45^, and its original assumption is that there is no significant difference between the variables to be tested and the level of significance $\alpha = 0.05$. The results of the K–W test are shown in Table 9.

**Table 9:** The *p*-values of the K-W test.

	Input variables	RMSE	NMSE	MAE	MAPE	NSCE
Kendall-τ-GWO-SVM vs Kendall-τ-WOA-SVM	T, Tmax, Tmin, P, WS	0.117	0.117	0.117	0.117	0.117
	T, Tmax, Tmin, WS	0.738	0.738	0.738	0.738	0.738
	T, Tmax, Tmin, P	0.071	0.071	0.071	0.071	0.071
	T, Tmax, Tmin	0.295	0.295	0.295	0.295	0.295
Kendall-τ-GWO-SVM vs Kendall-τ-GWO-LSTM	T, Tmax, Tmin, P, WS	0.009	0.009	0.009	0.009	0.009
	T, Tmax, Tmin, WS	0.009	0.009	0.009	0.015	0.007
	T, Tmax, Tmin, P	0.009	0.009	0.009	0.009	0.008
	T, Tmax, Tmin	0.009	0.009	0.009	0.009	0.007
Kendall-τ-GWO-SVM vs Kendall-τ-WOA-LSTM	T, Tmax, Tmin, P, WS	0.009	0.009	0.009	0.009	0.009
	T, Tmax, Tmin, WS	0.016	0.016	0.016	0.016	0.016
	T, Tmax, Tmin, P	0.009	0.009	0.009	0.009	0.009
	T, Tmax, Tmin	0.009	0.009	0.009	0.009	0.009
Kendall-τ-WOA-SVM vs Kendall-τ-GWO-LSTM	T, Tmax, Tmin, P, WS	0.009	0.009	0.009	0.009	0.009
	T, Tmax, Tmin, WS	0.016	0.016	0.016	0.016	0.016
	T, Tmax, Tmin, P	0.009	0.009	0.009	0.009	0.009
	T, Tmax, Tmin	0.009	0.009	0.009	0.009	0.009
Kendall-τ-WOA-SVM vs Kendall-τ-WOA-LSTM	T, Tmax, Tmin, P, WS	0.009	0.009	0.009	0.009	0.009
	T, Tmax, Tmin, WS	0.009	0.009	0.009	0.015	0.007
	T, Tmax, Tmin, P	0.009	0.009	0.009	0.009	0.008
	T, Tmax, Tmin	0.009	0.009	0.009	0.009	0.007
Kendall-τ-GWO-LSTM vs Kendall-τ-WOA-LSTM	T, Tmax, Tmin, P, WS	0.117	0.117	0.117	0.117	0.117
	T, Tmax, Tmin, WS	0.109	0.109	0.109	0.109	0.109
	T, Tmax, Tmin, P	0.251	0.251	0.251	0.251	0.251
	T, Tmax, Tmin	0.117	0.117	0.117	0.117	0.117

Table 9 shows that the *p*-values of the K–W test between the hybrid Kendall-τ-GWO-SVM and Kendall-τ-WOA-SVM models are all greater than 0.05, which means that there is no significant difference in the estimation accuracy of these two models with the same input combination; The p-values of the K-W test between shallow ML models and deep learning models are all less than 0.05, suggesting that there is a significant difference in the estimation accuracy under the same input combination; As for the hybrid Kendall-τ-GWO-LSTM and Kendall-τ-WOA-LSTM models, the *p*-values of K–W test are all greater than 0.05, suggesting that the model performance of these two models have little difference in the estimation of Ep with limited meteorological parameters.

To compare the model performance of the hybrid Kendall-τ-GWO-SVM, Kendall-τ-WOA-SVM, Kendall-τ-GWO-LSTM, and Kendall-τ-WOA-LSTM models, the performance indexes average in the testing stage were calculated, and shown in Table 10. It should be noted that the minimum verage of MAE, RMSE, MAPE, and the maximum average of NSCE were marked in bold. Table 10 shows that the minimum average of MAPE is 28.10% and the maximum average of NSCE is 0.77 when the input meteorological parameters of the hybrid Kendall-τ-WOA-SVM model are T, Tmax, Tmin, and P. Compared with the hybrid Kendall-τ-WOA-SVM model, the hybrid Kendall-τ-GWO-SVM model with the same input combination performed slightly better than the hybrid Kendall-τ-WOA-SVM model, the minimum average of MAPE is decreased from 28.10 to 27.03%, and the maximum average of NSCE is 0.77.

**Table 10:** The evaluation metrics average of the proposed models with different input combinations in testing stage.

Model	Input variables	RMSE	NMSE	MAE	MAPE	NSCE
Kendall-τ-WOA-SVM	T, Tmax, Tmin, P, WS	55.58	0.18	44.51	32.05%	0.73
	T, Tmax, Tmin, WS	56.32	0.23	48.23	37.56%	0.72
	T,T max, Tmin, P	**51.79**	**0.15**	**40.34**	**28.10%**	**0.77**
	T, Tmax, Tmin	52.61	0.18	43.42	32.38%	0.76
Kendall-τ-GWO-SVM	T, Tmax, Tmin, P, WS	55.38	0.17	43.89	31.37%	0.73
	T, T ax, Tmin, WS	56.60	0.22	47.78	36.53%	0.72
	T, Tmax, Tmin, P	**51.85**	**0.14**	**39.81**	**27.03%**	**0.77**
	T, Tmax, Tmin	52.90	0.18	43.60	32.56%	0.76
Kendall-τ-GWO-LSTM	T, Tmax, Tmin, P, WS	47.92	0.38	35.72	27.87%	0.80
	T, Tmax, Tmin, WS	50.07	0.44	36.98	29.54%	0.78
	T, Tmax, Tmin,P	41.53	0.28	31.69	24.58%	0.85
	T,T max, Tmin	**37.76**	**0.24**	**27.98**	**21.91%**	**0.88**
Kendall-τ-WOA-LSTM	T, Tmax, Tmin, P, WS	50.87	0.37	37.64	29.20%	0.77
	T, Tmax, Tmin, WS	56.13	0.47	42.99	32.97%	0.73
	T, Tmax, Tmin, P	42.95	0.27	32.85	24.65%	0.84
	T, Tmax, Tmin	**42.46**	**0.24**	**32.00**	**23.51%**	**0.84**

Although both the hybrid Kendall-τ-WOA-SVM and Kendall-τ-GWO-SVM models can be used to accurately simulate Ep with limited meteorological parameters, the estimation accuracy of these two models needs to be further improved since shallow ML models can not fully extract the nonlinear-and-dynamic-features between the meteorological parameters and Ep. As shown in Table 10, the minimum average MAPE of the hybrid Kendall-τ-GWO-LSTM and Kendall-τ-WOA-LSTM models are 21.91% and 23.51%, and the maximum average of NSCE are 0.87 and 0.84, implying that the estimating accuracy is significantly improved. Compared with Kendall-τ-GWO-SVM, the minimum average of MAPE decreased from 28.10 to 21.91%, and the maximum average of NSCE increased from 0.77 to 0.88, which means that the deep learning models significantly improved the estimating accuracy. In addition, the optimal and minimum input meteorological parameters of the hybrid Kendall-τ-GWO-LSTM and Kendall-τ-WOA-SVM models are T, Tmax, and Tmin, suggesting that deep learning models need fewer meteorological parameters to be observed than that of shallow ML models.

**Figure 7:**
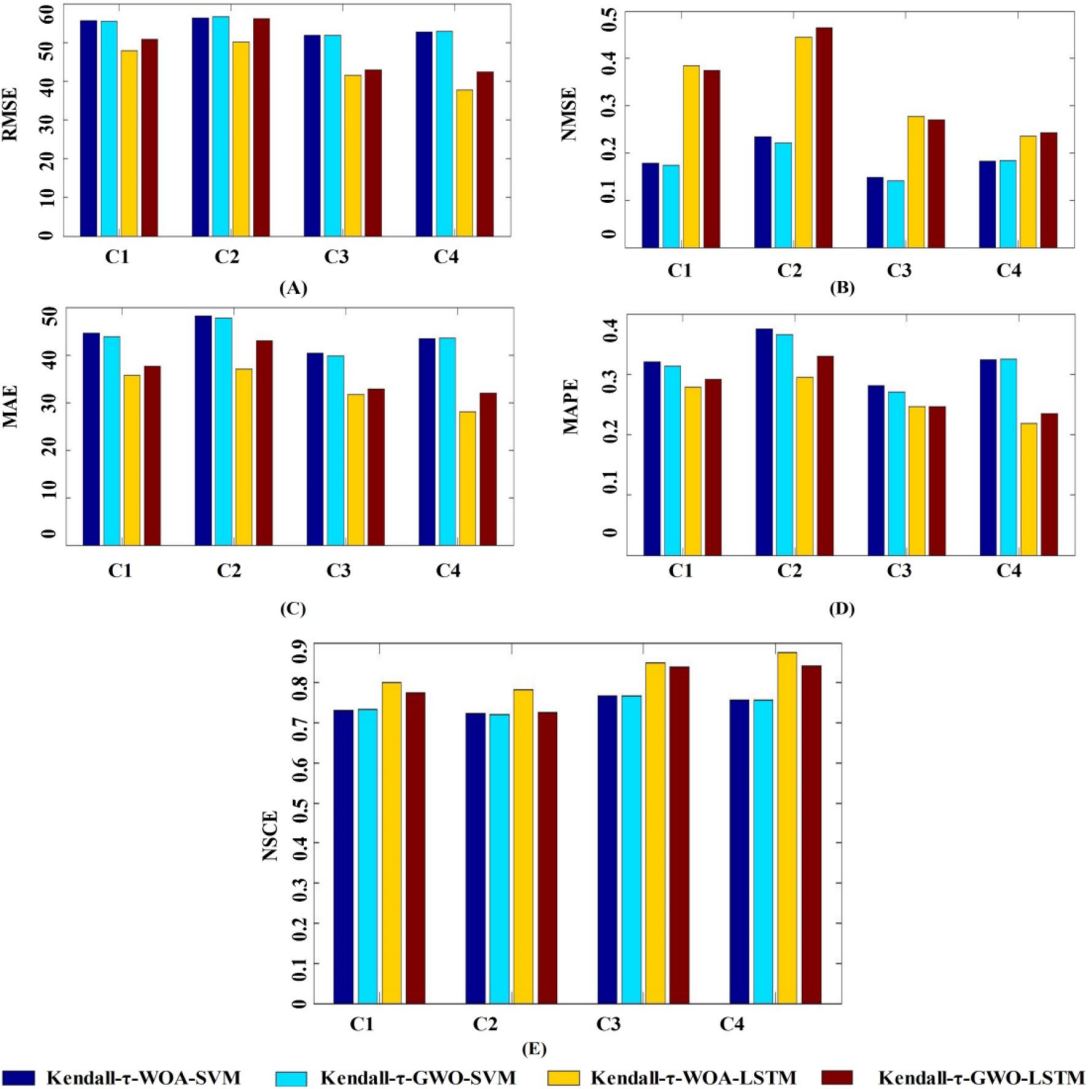
The performance indexes average of the proposed models with different input combinations. (**A**) RMSE. (**B**) NMSE. (**C**) MAE. (**D**) MAPE. (**E**) NSCE.

Figure 7 intuitively shows the performance indexes average of the proposed models with different input combinations. As shown in Fig. 7, the statistical metrics of the hybrid Kendall-τ-GWO-LSTM and Kendall-τ-WOA-LSTM models were similar to each other in the testing stage, suggesting that those two models can be employed to estimate Ep in dryland. Whereas, the negative evaluation indexes of Kendall-τ-GWO-LSTM are all smaller than that of Kendall-τ-WOA-LSTM, and NSCE showed the opposite trend, which means that the hybrid Kendall-τ-GWO-LSTM model performed better than the hybrid Kendall-τ-WOA-LSTM model, and GWO can obtain the optimal hyper-parameters of LSTM more effectively than WOA. Therefore, the hybrid Kendall-τ-GWO-LSTM model is strongly recommended to estimate Ep with limited meteorological parameters in dryland.

## Conclusion

In this study, four novel data-driven models, including the hybrid Kendall-τ-GWO-SVM, Kendall-τ-WOA-SVM, Kendall-τ-GWO-LSTM, and Kendall-τ-WOA-LSTM models, were proposed to estimate the monthly Ep with limited meteorological parameters, the proposed models simultaneously conduct the input meteorological variables and hyper-parameters optimization. The results illustrate that the optimal input meteorological parameters of the hybrid Kendall-τ-GWO-SVM (with C = 145.35 and G = 0.013) and Kendall-τ-WOA-SVM (with C = 339.44 and G = 0.013) models are T, Tmax, Tmin, and P, the minimum MAPE for both model is 26.97%, and the maximum NSCE is 0.77; the optimal input meteorological parameters of the hybrid Kendall-τ-GWO-LSTM (with NHL = 47, NHU = 93, E = 57, MBS = 20, and LR = 0.005) and Kendall-τ-WOA-LSTM (NHL = 63, NHU = 76, E = 46, MBS = 29, and LR = 0.005) models are T, Tmax, and Tmin, the minimum MAPE are 19.96% and 21.30%, and the maximum NSCE are 0.89 and 0.88, suggesting that Kendall-τ-GWO-LSTM is outperformed the Kendall-τ-GWO-SVM, Kendall-τ-WOA-SVM, and Kendall-τ-WOA-LSTM models, and needs fewer meteorological parameters to be observed. Therefore, the hybrid Kendall-τ-GWO-LSTM model can be highly recommended to estimate Ep without adequate meteorological parameters in dryland.

Although the deep learning models coupled with heuristic algorithms and data preprocessing techniques show fairly higher computing performance than the shallow ML models, the transferability of the proposed models to other locations need to be further tested. In addition, the main estimation modules are mainly focused on one or two ML models, and the estimation results inevitably have systematic overestimation or underestimation, which will inevitably lead to the risk of model selection. Further works will focus on constructing the combination model by integrating multiple ML models to obtain more robust estimating results in different bioclimatic zones.

## Supplementary Information

Supplementary Information.

## Data Availability

All data analyzed or generated during this study are included in the Supplementary Information.

## References

[1] Moazenzadeh R (2018). Coupling a firefly algorithm with support vector regression to predict evaporation in northern Iran. Eng. Appl. Comp. Fluid..

[2] Wu LF (2020). Hybrid extreme learning machine with meta-heuristic algorithms for monthly pan evaporation prediction. Comput. Electron. Agr..

[3] Li XR (2016). Fundamental Ecohydrology of Ecological Restoration and Recovery in Sand Desert Regions of China.

[4] Li XR (2018). Hydrological response of biological soil crusts to global warming: A ten year simulative study. Glob Change Biol..

[5] Wen X (2015). Support vector machine based models for modeling daily reference evapotranspiration with limited climatic data in extreme arid regions. Water Resour. Manag..

[6] Feng Y (2016). Comparison of ELM, GANN, WNN and empirical models for estimating reference evapotranspiration in humid region of Southwest China. J. Hydrol..

[7] Feng Y (2017). Evaluation of random forests and generalized regression neural networks for daily reference evapotranspiration modelling. Agr. Water Manag..

[8] Malik A, Kumar A, Kisi O (2018). Daily pan evaporation estimation using heuristic methods with gamma test. J. Irrig. Drain. Eng. ASCE..

[9] Rezaie-Balf M, Kisi O, Chua LH (2019). Application of ensemble empirical mode de composition based on machine learning methodologies in forecasting monthly pan evaporation. Hydrol. Res..

[10] Elbeltagi A (2022). Data intelligence and hybrid metaheuristic algorithms-based estimation of reference evapotranspiration. Appl. Water Sci..

[11] Elbeltagi A (2022). Modelling daily reference evapotranspiration based on stacking hybridization of ANN with meta-heuristic algorithms under diverse agro-climatic conditions. Stoch. Environ. Res. Risk. Assess..

[12] Fu TL, Li XR (2022). Hybrid the long short-term memory with whale optimization algorithm and variational mode decomposition for monthly evapotranspiration estimation. Sci. Rep..

[13] Fu TL (2021). A novel integrated method based on a machine learning model for estimating evapotranspiration in dryland. J. Hydrol..

[14] Kushwaha NL (2022). Evaluation of data-driven hybrid machine learning algorithms for modelling daily reference evapotranspiration. Atmos. Ocean.

[15] Fan JL (2019). Light gradient boosting machine: An efficient soft computing model for estimating daily reference evapotranspiration with local and external meteorological data. Agric. Water. Manag..

[16] Kushwaha NL (2021). Data intelligence model and meta-heuristic algorithms-based pan evaporation modelling in two different agro-climatic zones: A case study from Northern India. Atmosphere.

[17] Elbeltagi A (2023). Forecasting monthly pan evaporation using hybrid additive regression and data-driven models in a semi-arid environment. Appl. Water Sci..

[18] Pande CB (2022). Forecasting of SPI and meteorological drought based on the artificial neural network and M5P model tree. Land.

[19] Gocić M (2015). Soft computing approaches for forecasting reference evapotranspiration. Comput. Electron. Agric..

[20] Jain SK, Nayak PC, Sudheer KP (2008). Models for estimating evapotranspiration using artificial neural networks, and their physical interpretation. Hydrol. Process..

[21] Petković D (2016). Particle swarm optimization-based radial basis function network for estimation of reference evapotranspiration. Theor. Appl. Climatol..

[22] Anurag M (2022). Deep learning versus gradient boosting machine for pan evaporation prediction. Eng. Appl. Comp. Fluid..

[23] Shrestha NK, Shukla S (2015). Support vector machine based modeling of evapotranspiration using hydro-climatic variables in a sub-tropical environment. Agric. Forest Metoorol..

[24] Fan JL (2018). Evaluation of SVM, ELM and four tree-based ensemble models for predicting daily reference evapotranspiration using limited meteorological data in different climates of China. Agr. Forest Metoorol..

[25] Rezaie-balf M (2017). Wavelet coupled MARS and M5 Model Tree approaches for groundwater level forecasting. J. Hydrol..

[26] Aghajanloo MB, Sabziparvar AA, Hosseinzadeh TP (2013). Artifificial neural network-genetic algorithm for estimation of crop evapotranspiration in a semiarid region of Iran. Neural Comput. Appl..

[27] Kim S (2008). Neural networks and genetic algorithm approach for nonlinear evaporation and evapotranspiration modeling. J. Hydrol..

[28] Zhu B (2020). Hybrid particle swarm optimization with extreme learning machine for daily reference evapotranspiration prediction from limited climatic data. Comput. Electron. Agric..

[29] Mohammadi B, Mehdizadeh S (2020). Modeling daily reference evapotranspiration via a novel approach based on support vector regression coupled with whale optimization algorithm. Agric. Water Manag..

[30] Farshad A (2021). Application of an artificial intelligence technique enhanced with intelligent water drops for monthly reference evapotranspiration estimation. Agric. Water. Manag..

[31] Saray MH (2020). Regionalization of potential evapotranspiration using a modified region of influence. Theor. Appl. Climatol..

[32] Kisi O, Alizamir M (2018). Modelling reference evapotranspiration using a new wavelet conjunction heuristic method: wavelet extreme learning machine vs wavelet neural networks. Agric. Forest Metoorol..

[33] Abdullah SS (2015). Extreme learning machines: a new approach for prediction of reference evapotranspiration. J. Hydrol..

[34] Karbasi M (2018). Forecasting of multi-step ahead reference evapotranspiration using wavelet- gaussian process regression model. Water Resour. Manag..

[35] Dinesh KV (2022). Methods to estimate evapotranspiration in humid and subtropical climate conditions. Agric. Water Manag..

[36] Granata F, Di Nunno F (2021). Forecasting evapotranspiration in different climates using ensembles of recurrent neural networks. Agric. Water Manag..

[37] Chen ZJ (2020). Estimating daily reference evapotranspiration based on limited meteorological data using deep learning and classical machine learning methods. J. Hydrol..

[38] Majhi B (2019). Improved prediction of daily pan evaporation using deep-LSTM model. Neural Comput. Appl..

[39] Solé R, Levin S (2022). Ecological complexity and the biosphere: The next 30 years. Philos. Trans. R. Soc. B..

[40] Hochreiter S, Schmidhuber J (1997). Long short-term memory. Neural Comput..

[41] Zuo G (2020). Decomposition ensemble model based on variational mode decomposition and long short-term memory for streamflow forecasting. J. Hydrol..

[42] Vapnik V (1998). Statistical Learning Theory.

[43] Mirjalili S, Mirjalili SM, Lewis A (2014). Grey Wolf optimizer. Adv. Eng. Softw..

[44] Mirjalili S, Lewis A (2016). The whale optimization algorithm. Adv. Eng. Softw..

[45] McDonald JH (2014). Handbook of Biological Statistics.

